# Nutritional enhancement of animal feed and forage crops via genetic modification

**DOI:** 10.1080/03036758.2024.2387136

**Published:** 2024-08-08

**Authors:** Gregory Bryan, Somrutai Winichayakul, Nick Roberts

**Affiliations:** AgResearch Limited, Grasslands Research Centre, Palmerston North, New Zealand

**Keywords:** Genetic modification, plant biotechnology, white clover, perennial ryegrass, metabolisable energy, commercialisation

## Abstract

Introducing beneficial productivity and nutritional traits into food, feed and forage crops utilising the tools of biotechnology can lead to improvements via genetic modification that cannot be achieved by traditional plant breeding. The timelines and costs are significant, and the regulatory hurdles can lead to some promising traits failing to be commercialised. These challenges mean that large commodity crops are the primary beneficiaries of biotechnology. New Zealand being primarily a grazing pastoral agricultural market and small in international terms, faces greater challenges. The species used in pastoral agriculture have relatively small seed markets and therefore limited investment for genetic improvement. The nutritional quality of feed and forages both in composition and in energy density per ha has a major influence on animal productivity and the environmental impacts of agriculture. Through government and private industry support, AgResearch has developed novel approaches to improve plant photosynthesis, energy density, and nutritional quality that has applications in crops globally. This review discusses the different challenges and solutions to improving plant nutrient density and outlines the benefits of these novel biotechnology traits in animal forage and feed crops.

## Introduction

While genetically modified (transgenic) crops have been grown commercially for nearly 30 years and are grown in 28 countries on 180 m ha, there are no genetically modified crops grown commercially in New Zealand. The first genetically modified crops developed and commercialised in the USA were corn and soybean, containing input traits for herbicide resistance and insect resistance. These traits primarily benefit the farmer and improve farm efficiency, reduce herbicide use and protect productivity. Traits that benefit consumers have taken much longer to reach the market and the reasons for this are complex. A major hurdle has been the cost of developing genetically modified plant species that translate from the lab to the field with no impact on crop yield and the significant regulatory approval cost. Crops such as corn, soybean and canola are planted annually at large scale (37–40 m ha in the USA for both soybean and corn), therefore there is a large annual seed market creating significant value for the large seed companies. This enables significant reinvestment into crop improvement using traditional breeding methods, marker assisted breeding, gene editing and genetic modification. Traditionally, genetically modified crops were the domain of the large agricultural biotechnology companies, although this is changing in recent times.

New Zealand is primarily a pastoral agriculture economy with significant horticulture and forestry contributions to the economy. The Dairy industry contributes $17 billion of exports to New Zealand’s economy annually, with 20.7 billion litres of milk produced in 2020 from approximately 5.9 m dairy cattle on 2.44 m ha of dairy pasture. There are also 3.9 m beef cattle on 2.38 m ha of land, 26 m sheep on 8.7 m ha of land and 0.8 m deer on 0.26 m ha of land and these three species contribute $8.5 billion of meat exports (Beef and Lamb 2020). The horticulture industry reported $4.6 billion of exports in 2021/22 and the value of the industry was $7 billion (Horticulture NZ 2022). New Zealand forestry exports were valued at $6.64 billion in 2022 (MPI 2022).

The most important pasture crops are perennial ryegrass (*Lolium perenne* L.) and the legume white clover (*Trifolium repens* L.) and are grown on approximately 6.24 million ha. There are a range of alternative species on hill country farmland, but the main annual seed markets are perennial ryegrass and white clover. Internationally perennial ryegrass and white clover are important in temperate regions utilising grazing pastoral agriculture.

The impacts of growing and utilising genetically modified crops and forages for New Zealand has been recently reviewed by Caradus (2023). The review examined consumer attitudes to food produced from genetically modified plants or animals fed genetically modified plants and whether this would reduce demand for New Zealand products if genetic modification was adopted. It was found most consumers are driven by price and not the method of production, although the method of modification has some importance. It was concluded adoption of genetically modified crops would have no long-term deleterious effects in overseas markets. The recently elected (2023) New Zealand government has stated it will review the current HSNO Act (1996) and introduce new regulations to help New Zealand move forward in benefiting from genetically modified crops.

Nutritionally enhanced food crops are available in international markets and there are detailed reviews of these including many that are still in development (Hefferon 2015; Scharff et al. 2022). This review focusses on genetically modified animal feed and forage crops which currently have few innovations available in international markets and looks at this from a New Zealand perspective with regards to our important pastoral agriculture industry. While traditional plant breeding is the underpinning method for crop improvement, the improvement of nutritional quality is a challenging target and relatively small improvements have been made over many years. In some cases, the breeding incentives for increased seed yield while highly successful, have come at the expense of nutritional quality, for example in soybean where seed protein content has declined as yields increased (Froes de Borja Reis et al. 2020). The value of genetic modification is the ability to make a step-change in nutritional quality by introducing new traits or removing antinutritional properties in forages and this can be superimposed on top of enhancements from traditional breeding. The first and only genetically modified animal forage/feed crop with nutritional enhancement commercialised internationally was a reduced lignin alfalfa variety released in the USA in 2015. Several products are in development in forages and feed crops and these are discussed. Two technologies in development by New Zealand science teams are white clover with foliar condensed tannins (High Condensed Tannin (HiCT)) white clover and perennial ryegrass with increased metabolisable energy (High Metabolisable Energy (HME)) perennial ryegrass. Both are expected to improve animal nutrition and reduce the environmental impacts of pastoral farming. The two technologies are complementary and could both be utilised in New Zealand pastures. These innovations are examined in relation to other approaches to improving feed and forage nutritional quality.

The high metabolisable energy technology under development for perennial ryegrass was aimed at increasing metabolisable energy by accumulating foliar lipids. However, a serendipitous discovery that this technology also increased plant photosynthesis has provided opportunities in other crops (www.zeakal.com). The detailed and innovative scientific research surrounding this technology and its application in forages and animal feed crops are discussed.

## Improving nutritional quality of forage crops

### Condensed tannins in white clover

Forage legumes such as white clover, generally grown in mixed swards with forage grasses, and alfalfa or lucerne (*Medicago sativa* L.), grown as a single species sward provide value to animal production due to their high protein content and additionally are important atmospheric nitrogen fixers in pastures. However, during digestion in the rumen, nitrogen losses from excreted ammonia can occur (Pacheco and Waghorn 2008) and the rapid intake of highly soluble proteins can increase the susceptibility to bloat (Ledgard et al. 1990). Condensed tannins (CTs) bind to protein to form insoluble complexes that increase resistance to degradation by rumen microbes. These complexes disassociate as they pass through the acidic abomasum and the released proteins can be absorbed in the animal’s small intestine (Jones and Mangan 1977).

Several forage legume species contain CTs e.g. sanfoin (*Onobrychis viciiifolia* L.), sulla (*Hedysarum coronarium* L.) and lotus (*Lotus corniculatus* L.) These are not agronomically compatible with Australasia’s pastoral grazing system (Roldan et al. 2020). White clover contains condensed tannins in the flowers and leaf trichomes but the overall concentration within the leaves is insufficient to provide benefit to grazing ruminants. Breeders have attempted to use conventional breeding techniques to develop white clover with foliar condensed tannins and have been unsuccessful. Cultivars with a greater concentration of floral CTs, with a longer flowering period and a higher density of flowers have been successfully bred. However, they have not successfully been utilised to provide greater CTs in ruminant diets mainly due to agronomic reasons and it was concluded that the incidence of flowering as a source of CTs was unlikely to improve the nutritive value of white clover (Burggraaf et al. 2004; 2008).

Genetic modification has successfully been used to develop white clover with foliar CTs (Woodfield et al. 2019; Roldan et al. 2020; 2022; Caradus et al. 2022). A transcription factor isolated from a white clover relative hare’s-foot clover (*T. arvense*) with foliar CTs was introduced into white clover. This resulted in production of CT in white clover leaves of 1.2% (of DW). The CTs predominantly of prodelphinidins and procyanidins and importantly had a mean polymerisation of 10 flavan-3-ol subunits (required for binding of proteins).

To validate the efficacy of the CTs in genetically modified white clover, *in vitro* rumen assays were performed (Roldan et al. 2022). The study demonstrated a reduction of methane emitted by 19% compared to non-CT white clover and ammonia production was reduced by 60%. *In vivo* studies will ultimately be required to demonstrate the efficacy of this trait and the *in vitro* assays have provided valuable insight and inform animal trial design.

Breeding the HiCT trait into elite white clover cultivars is an important process to develop commercial cultivars. An outline of some of the early stages of a breeding programme is outlined in Woodfield et al. (2019). It was found that crossing the trait into high anthocyanin lines increased the level of foliar condensed tannins threefold (Roldan et al. 2020). The levels of CTs were stable over generations and the CT levels measured in a containment facility successfully translated into regulated field trials in the USA.

Regulated field trials have been initiated in Australia in 2023 under the Office of the Gene Technology Regulator (OGTR) and this provides an opportunity to assess plant agronomic traits in the field and progress the breeding programme. This promising technology offers benefits for grazing ruminant nutrition and reduction of the environmental impacts of nitrogen excretion and ruminant methane emissions. There is the potential to utilise HiCT white clover alongside high metabolisable energy perennial ryegrass (discussed later in this article). As both technologies offer potential production and environmental benefits, it will be important to assess both HiCT white clover and HME perennial ryegrass individually and in combination in detailed animal nutrition trials.

### Reduced lignin in alfalfa and forage grasses

Lignin is a polymeric macromolecule and major component of the cell wall in vascular plants. It is multifunctional providing structural support for plant cells and tissues (Boerjan et al. 2003), protection against pathogens (Hammond-Kosack and Jones 1996) and enhances the hydrophobicity of plant vasculature (Kubitzki 1987). The link between cell wall composition including lignin concentration on digestibility has been well studied including in alfalfa (Reddy et al. 2005) and the forage grass perennial ryegrass (Colas et al. 2022) and there has been significant effort made to characterise the biochemical pathway of lignin biosynthesis in forages such as perennial ryegrass (Louie et al. 2010).

Alfalfa is an important perennial forage legume grown in temperate regions of the world. There are 30 m ha grown world-wide with USA, Argentina and Canada being the main producers. Alfalfa has several environmental benefits. It is a nitrogen fixer, has a root system that can access deep water within the soil profile, and it can yield multiple harvests over several seasons.

The main limitation to nutritive value is the polyphenolic lignification of the cell walls in the stems which can compose 50–70% of the total plant biomass. Breeders have developed reduced lignin cultivars primarily by changing the leaf to stem ratio and breeding for stem cell wall digestibility which correlates with lower lignin concentration. There is a correlation between lower lignin concentrations and fibre digestibility (Reddy et al. 2005). It has been shown that increased neutral detergent fibre (NDF) digestibility *in vitro* correlates with increased dry matter intake and increased milk production in dairy cows (Oba and Allen 1999). Genetic modification has been used to down regulate genes in the lignin biosynthetic pathway. Barros et al. (2019) reported on the down regulation of the gene encoding caffeol-CoA 3-*O*-methyltransferase (CCOMT). These plants have approximately 15% less lignin than control biotypes and increased NDF digestibility. Karls et al. (2022) performed nutrition trials on growing beef steers and compared three alfalfa biotypes, a control biotype, genetically modified low lignin biotype and a biotype with a high leaf to stem ratio. The genetically modified low lignin biotype had the highest NFD digestibility. However, they found that there was no effect on dry matter intake or daily weight gain by growing steers. The genetically modified reduced lignin alfalfa has been shown to have a 4.8–7.0% lower dry matter yield than conventional alfalfa (Arnold et al. 2019). These outcomes highlight the complexity of altering lignin concentration in alfalfa and potentially other forage crops via conventional breeding or genetic modification. While there is positive outcome for milk production in dairy cows from lower lignin in forages this does not necessarily translate into increase dry matter intake and daily weight gain in beef steers.

The C*COMT* and *Cinnamoyl-CoA-reductase* (CCR) genes from perennial ryegrass were down regulated and field grown plants in regulated trials had 37% reductions for CCR downregulated plants and 6% reductions in lignin for COMT downregulated plants (Tu et al. 2010). There was no difference in plant vigor in cooler winter temperatures and in hot conditions plant vigor was impacted. The CCOMT downregulated plants had greater susceptibility to rust (*Puccinia* spp.). The potential digestibility of plant samples was assessed by near infra-red spectroscopy and the CCR and CCOMT downregulated plants had higher digestibility scores than the control lines. The study helped to improve understanding of the complex phenolpropanoid metabolic pathway. There have also been attempts to reduce lignin concentration in the C4 photosynthetic *Paspalum dilatatum* where the *CCR* gene was downregulated leading to a 7.5-18.4% reduction in lignin in leaf blades (Giordano et al. 2014). There are no reports of animal feeding trials for these grasses so the benefits for animal nutrition are not confirmed.

### Increased water soluble carbohydrates in forage grasses

Fructans are water soluble carbohydrate polymers of fructose and glucose and are short – and long – term carbohydrate reserves in temperate forage grasses. Attempts have been made to increase fructans by traditional plant breeding and genetic modification. Overexpression of two genes encoding *6-glucose fructosyltransferase* and *sucrose:sucrose 1-fructosyl-transferase* led to plants with fructan accumulation of the leaves and at peak levels would add 1.7 MJ/kg of metabolisable energy (Panter et al. 2017; Badenhorst et al. 2018). When photosynthetic promoters were used to regulate expression of these genes some plants had increased biomass when grown in containment conditions (Panter et al. 2017). While no mechanism was suggested, we hypothesise it could be due to a reduction in the negative feedback of photosynthesis. No genetically modified high fructan lines have been commercialised to date.

### High metabolisable energy forages

Through long term government and private industry financial support, AgResearch has adopted a novel approach to improve plant photosynthesis and energy density, that has applications in food and feed crops globally. The productivity of ruminants on grass diets is constrained by the inefficient conversion of plant energy and protein into animal products (Kingston-Smith and Theodorou 2000). In the early 2000s, an assessment was made that accumulating fatty acids in forages was a viable approach to improve the nutritional density of forages. Plant lipids contain twice the calorific energy of carbohydrates and protein per unit of weight. It was hypothesised that increasing the total lipid concentration of forages may increase energy intake and animal productivity. This was the genesis of high metabolisable energy (HME) perennial ryegrass.

To validate the concept of increased lipid composition of forage grasses a study was conducted in weaned lambs grazing on ryegrass (containing 4% total lipids DW) where they received twice daily oral doses of a blend of 75% linseed and 25% sunflower oil at 0, 28 and 56 ml/day (Cosgrove et al. 2004). This supplementation simulated diets of low (4%), medium (6%), and high (8%) total lipid respectively. No difference in liveweight gain was detected but lambs eating the high (8%) total lipid diet ate 16% less dry matter and had a 33% higher feed conversion efficiency. Blood plasma and carcass meat from lambs supplemented with plant oils had lower concentrations of short chain and saturated fatty acids and higher concentrations of poly-unsaturated fatty acids. This suggests that meat from animals consuming greater quantities of unsaturated fatty acids may have human health attributes. Additional support for increasing lipids in perennial ryegrass comes from the modelling and decision support tool FARMAX® (Bryant et al. 2010), where the model was used to simulate a farm with perennial ryegrass containing 1 MJ/kg DM greater metabolisable energy. The model assessed three different stocking rates and indicated that a 12-17% increase in milk solids production and a corresponding 6-7% decrease in nitrogen excretion was possible (unpublished data).

A detailed analysis of perennial ryegrass fatty acid content in several varieties over different seasons revealed that the seasonal variation of plant fatty acid content was greater than the difference between cultivars (unpublished data). At this stage there was limited data on genetic differences in perennial ryegrass fatty acid content and it was assumed that traditional plant breeding would not lead to significant increases in plant fatty acid content. More recent research (Hegarty et al. 2013) on perennial ryegrass fatty acid composition traits confirmed that differences in fatty acid content were largely determined by seasonal factors rather than by differences in plant varieties. Twenty-one quantitative trait loci (QTL) were mapped onto a ryegrass genetic map with individual QTLs contributing between 8.5 and 20.2% of the total genetic variance. Different sampling dates explained 75-91% of the variance in individual fatty acid content whereas the genotype explained between 9 and 19%. This finding supported the value of utilising modern biotechnology tools such as genetic modification to increase fatty acid content.

#### Development of high metabolisable energy perennial ryegrass

An obvious fatty acid biosynthesis gene target was the enzyme responsible for the last and only committed step in triacylglycerol (TAG) biosynthesis, diacylglycerol *O*-acyltransferase (DGAT1). In plant leaves, TAG is a short-term storage intermediate of thylakoid lipid in the formation and turnover of cell membranes (Troncoso-Ponce et al. 2013). Several research groups had attempted to increase leaf TAG levels by overexpression of *DGAT1* and other genes involved in fatty acid biosynthesis in plant leaves, only to find the TAG was catabolised by plant lipases (Winichayakul et al. 2008). In some cases, the result has been growth penalties due to pleiotropic effects or due to the diversion of carbon into increased lipids (Vanhercke et al. 2019). Plant seeds protect TAG by encapsulating lipid droplets with a protein called oleosin (Tzen et al. 1992). The AgResearch team had been investigating modifications to oleosin for expression in microorganisms and plants and had tested multiple oleosin units joined in tandem head-to-tail fusions (termed polyoleosin) in transgenic arabidopsis (Scott et al. 2010). While this was successfully expressed in seeds and leaves, further innovation was required to develop a suitable lipid protecting molecule in plant leaves for stable long-term storage of lipids.

A critical breakthrough for stably increasing leaf lipids was the invention of Cysteine Oleosin (Cys-Ole), a modification of the oleosin molecule (Roberts et al. 2010; 2011; Winichayakul et al. 2013). An oleosin from *Sesame indicum* was engineered to contain different numbers of strategically placed cysteine residues with the goal of producing cross linked oleosins to increase stability of lipid droplets *in planta*. The concept was first tested in yeast and in the transgenic model plant species arabidopsis (Winichayakul et al. 2013). In arabidopsis it was found that neither DGAT1 alone nor DGAT1 with native sesame oleosin constitutively expressed in plant tissues significantly increased leaf lipid levels in leaves harvested at 35 days. However, one version of Cys-Ole (with three cysteine substitutions in each amphipathic arm of the molecule), co-expressed with DGAT1, led to the long – term accumulation of plant fatty acids and the accumulation of triacylglycerol in the leaves and roots of transgenic arabidopsis, effectively doubling leaf TAG levels in leaves harvested at 35 days.

Unexpectedly, the fatty acid accumulation also correlated with a 50% increase in leaf biomass and 24% increase in the CO_2_ assimilation rate per unit leaf area (an increase in plant photosynthesis). It has previously been hypothesised by Durrett et al. (2008) that increasing *de novo* fatty acid biosynthesis could lead to an increase in CO_2_ assimilation due to CO_2_ recycling. This was supported by the results in Winichayakul et al. (2013) although as discussed shortly, it is only part of the explanation for the increased photosynthesis. This serendipitous discovery has led to significant opportunities for a better understanding of the regulation of photosynthesis and for application in other plant species.

The next step was to co-express Cys-Ole and DGAT1 in perennial ryegrass, and a further enhancement of the technology was to utilise photosynthetic regulatory elements to obtain expression in the plant green tissues. It was considered that the accumulation of fatty acids in the plant roots was unnecessary and would be energetically costly. This led to the first HME prototype perennial ryegrass plants and increases in leaf lipids of nearly double (6-7% of DW) the levels present in conventional perennial ryegrass plants (Beechy-Gradwell et al. 2018; Beechy-Gradwell et al. 2020; Cooney et al. 2021). Many attempts to alter or enhance plant attributes using genetic modification have shown promise in glasshouse or controlled environment conditions only to have either lacked effective translation into the field or the plants have had significant yield penalties. Part of the strategy for minimising these risks is to conduct detailed research on plant performance, physiology, biochemistry in both the lab and the field (discussed in the next section).

The response to different levels of added nitrogen, water use and growth under water limiting conditions was assessed in experiments where plants were propagated as spaced plants in pots under controlled environment conditions (Beechy-Gradwell et al. 2018). A cutting regime that simulated grazing was utilised, and plant regrowth was greater in HME perennial ryegrass compared to the wild-type ryegrass an observation consistent to arabidopsis containing the same technology. Growth of HME perennial ryegrass (shoot DW) was greater than wild type when supplied nitrate, ammonia or urea with nitrogen use efficiency greater in elevated levels. Leaf total fatty acid levels (% of DW) increased in both wild type and HME perennial ryegrass with increasing nitrate application although HME perennial ryegrass leaves were consistently 50% higher than wild type, primarily through increases in C18:1 and C18:2 fatty acids. The conclusion was that nitrogen supply rather than nitrogen form was of primary importance for HME perennial ryegrass growth and fatty acid content and nitrogen use efficiency (NUE) was greater in high nitrogen loads. Water use was greater in HME perennial ryegrass driven primarily by the greater plant size. Water use efficiency (WUE) in HME perennial ryegrass was 16% higher than the wild type under both limiting and non-limiting water supply. The overall conclusion was if these benefits translated into the field HME perennial ryegrass could deliver the benefits of increased plant lipids with increased NUE and WUE.

The mechanism for increased photosynthesis was further elaborated in controlled environment experiments (Beechy-Gradwell et al. 2020) where photosynthesis, the relative growth rate and specific leaf area were evaluated under different nitrogen sources and ambient (400 ppm) and elevated (760 ppm) of CO_2_. It was speculated by the authors that by behaving as uniquely stable micro-sinks for carbon, Cys-Ole encapsulated lipid droplets can reduce feedback inhibition of photosynthesis and drive greater carbon capture. This has implications for other crop applications as it is potentially an approach to reduce the negative feedback of photosynthesis. Paul and Eastmond (2020) commented that the original hypothesis of CO_2_ recycling in Arabidopsis (Winichayakul et al. 2013) had been ruled out and as an alternative, the diversion of carbohydrate into lipid carbon sink sequesters carbon away from carbon-sensing mechanisms. This could mitigate the signals that would normally down regulate photosynthesis as part of carbon and energy metabolic homeostasis meaning that photosynthesis is ‘blind’ to carbon accumulation and can carry on unimpeded.

The relationships between photosynthesis, leaf nitrogen and the engineered micro-sinks for carbon were examined by Cooney et al. (2021). They identified a correlation with DGAT1 and cysteine oleosin accumulation, the increase in leaf fatty acids and increased photosynthesis per leaf unit area (A_area_). Plant lines with the largest increases did so at the expense of leaf sugar. It was determined that the novel carbon sink in the leaves of perennial ryegrass can induce leaf level changes which increase both A_area_ and photosynthetic nitrogen use efficiency. They determined that the content of the key enzyme in photosynthesis rubisco was unchanged and there was an increase in the specific leaf area, stomatal and mesophyll conductance and leaf nitrogen allocated to photosynthetic electron transport. Essentially HME perennial ryegrass has an increased efficiency of CO_2_ capture, transport and photosynthesis. This increased understanding of the mechanism for enhanced photosynthesis has implications for multiple crop applications in both C3 and C4 photosynthesis pathways for both human and animal feed.

Further understanding of the mechanisms and metabolic trade-offs in HME perennial ryegrass was provided in a study examining the regulatory networks of HME ryegrass under different irradiances (Winichayakul et al. 2022). They determined that co-expression of DGAT1 and Cys-Ole promoted carbon flux to fatty acid synthesis in leaves as there was an increase in transcripts involved in plastid *de novo* fatty acid biosynthesis. Interestingly, the overall fatty acid content and transcription profiles of selected genes involved in lipid metabolism were not reduced or downregulated in response to low light. This suggests that even under light limiting conditions such as in a competitive pasture sward, where plants compete for light, the higher metabolisable energy content is maintained.

#### Translation from laboratory to field

The ultimate demonstration of the value of a novel genetic technology in crops is the effective translation from the glasshouse or controlled environment into the field. Many attempts at improving crops have failed at this stage as plants are exposed to competition for light and nutrients, face environmental stresses and exposure to pests and pathogens. The regulatory framework under the HSNO Act 1996 had provided a major hurdle for regulated field trials of genetically modified plants in New Zealand. One of the challenges is the risk-benefit analysis which creates something of a ‘Catch 22’ situation where field trials are needed to assess potential benefits but a lack of data on the benefits creates a barrier to receiving approval for field trials. For this reason, AgResearch and its stakeholders conducted five years of regulated field trials in Missouri, USA with USDA-APHIS approval.

Beechey-Gradwell et al. (2022), tested different HME perennial ryegrass populations consisting of null controls, and hemizygous HME genotypes in mini-swards over two seasons (2019 and 2020) with regular cutting to simulate grazing. A comparison was made with indoor growth conditions in New Zealand and field conditions in Missouri where the environment at the field trial site was generally hotter than a New Zealand summer. Translation from the lab to the field of the primary traits of leaf lipid content and gross energy were demonstrated. In containment the hemizygous HME ryegrass leaf fatty acid levels were 32% higher than the null controls and gross energy increased 0.2-0.5 kJ/gDW over the null control. In the field the hemizygous HME ryegrass leaf fatty acid levels were 25-34% higher than the null controls and gross energy increased 0.2-0.5 kJ/gDW over the null control. The intrinsic growth advantage of HME perennial ryegrass appeared diminished in field grown swards suggesting the benefits may only be realised under limited conditions.

Richardson et al. (2023) compared the growth and stability of the perennial ryegrass *Epichloë* fungal endophyte in both controlled environment and field conditions. Many perennial ryegrass cultivars contain commercial mutualistic fungal endophytes that increase the agronomic competitiveness of its host (Caradus et al. 2021). The benefits are achieved through *in planta* production of fungal secondary metabolites that vary depending on the strain utilised including Indole diterpenes and epoxyjanthitrems, which provide protection against a range of insect pests. As the perennial ryegrass plant and endophyte are in a mutualistic interaction, it is important to assess the impacts of any metabolic changes in the plant host due to the HME trait, on the stability of this interaction and on the production of protective secondary metabolites. These endophytes are seed transmitted and upon germination the fungus spreads systemically throughout the areal portions of the plant. The *Epichloë* derived secondary metabolites generally accumulate to higher concentrations in the seeds than in vegetative tissue which is thought to be a strategy to protect the germinating seedling as they translocate into the seedlings. Therefore, it is essential to determine that mycelial biomass and secondary metabolite production fall within the range expected for non-genetically modified commercial cultivars.

The study assessed both the HME trait and secondary metabolite production of two commercial endophytes AR1 and AR37 in controlled environments and in the field in Missouri. No differences in fungal transmission, fungal biomass, leaf fatty acid levels or plant biomass were observed between HME endophyte infected and endophyte free perennial ryegrass lines. The levels of fungal secondary metabolites were within the range previously measured in commercial cultivars. Overall, these results indicate that the mutualistic endophyte has no effect on the HME trait and plant growth in perennial ryegrass. In addition, the HME trait has no significant impact on endophyte transmission and mycelial biomass. These results demonstrated successful translation from the laboratory to the field for the important HME perennial ryegrass + endophyte mutualistic interaction.

#### Environmental benefits of HME perennial ryegrass

Methane emissions from ruminant livestock contribute to global anthropogenic emissions of greenhouse gasses. Several interventions are under development to reduce methane emissions from ruminant livestock including vaccines, inhibitors, low emission animals, alternative forages, HiCT white clover and HME perennial ryegrass.

Dietary fats have been demonstrated to reduce methane emissions from ruminants. A meta-analysis by Grainger and Beauchemin (2011) has found an inverse proportional relationship between methane emissions and increasing dietary fat. For each 1% increase in dietary fat there is a corresponding 5% reduction in methane emitted. The increase in total fatty acids in HME ryegrass (to 6-7% of plant DW) are in the range where methane reductions may be realised without impacting on animal health or creating milk fat depression in dairy cows.

To provide further support for the impact of HME perennial ryegrass for delivery of increased dietary fat, an *in vitro* gas production and rumen fermentation study was undertaken (Winichayakul et al. 2020). The study utilised ensiled HME and control perennial ryegrass as it has been found that the total fatty acid content is preserved during the ensiling process and the nutritional composition of ensiled material largely reflects those of fresh counterparts. Three different HME ryegrass genotypes and wild-type controls were prepared as fresh and as silage. Total fat in fresh and ensiled wild-type controls ranged from 3.54–3.77% total fatty acids (% of DM) whereas the HME ryegrass fresh and ensiled HME ryegrass ranged from 5.85–7.24% total fatty acids (% of DM). A 10-15% decrease in methane proportion of the total gas production was measured for HME perennial ryegrass. This reduction is consistent with the reductions reported in the Grainger and Beauchemin (2011) meta-analysis. The *in vitro* gas analysis is not a confirmation that HME ryegrass will reduce methane emissions from grazing ruminants, but it does help inform future animal trials and adds to the evidence of potential benefit. Confirmation will require methane emission research on both sheep and cattle. This will be a significant undertaking as the area of HME and control perennial ryegrass to perform a study in cattle could be several ha. It is likely the pasture will need to be grown over two seasons to allow establishment of the pasture prior to harvesting and ensiling the grass. This means managing plant reproduction to prevent pollen release will be required. Alternatively, a larger planting over one season could occur. The trade-off is the generation of sufficient seed in containment for the pasture plots. Depending on the method of planting, between 10 and 20 kg of seed is required. These challenges have provided hurdles for both the regulators in both New Zealand and Australia and on the logistics of such a trial.

### Cysteine oleosin and DGAT1 in animal feed crops

#### Soybean

Soybean is the most important oilseed and crop for the animal industry due to its high protein concentration and high abundance of essential and non-essential amino acids. Soybean is grown on 37 m ha annually in the USA and on a similar area in South America and provides approximately 70% of the worlds plant-based meal for animals. As the crop has been bred for increased yield, this has come at the expense of seed protein content. Since 1980 seed yield has increased 50%. However, protein content has declined 1.2% (Froes de Borja Reis et al. 2020). This has now become an issue for the industry in the USA as the protein content of hexane soybean meal is now at an all-time low and is near the minimal level for the industry standard. Any technology that increases protein content without impacting on crop yield will provide tremendous value for the industry. In addition, maintaining oil content adds tremendous value due to the increase in the renewable biodiesel market.

By developing a spin out biotechnology model with partners in the USA, the technology portfolio has been expanded to include further innovations: novel oil synthesising enzymes and plant architecture improvements. With their partners ZeaKal (www.zeakal.com) a venture capital backed company, AgResearch has developed a comprehensive intellectual property portfolio covering three biotechnology traits with 92 patents filed in multiple jurisdictions. This has enabled Zeakal to raise over US$30M of venture capital to develop soybean, hemp and corn.

The technology is trademarked as PhotoSeed™ and has been extensively tested over multiple years of regulated field trials. On 7th December 2022 the company obtained USDA-APHIS Regulatory Status Review approval (USDA-APHIS 2024) for PhotoSeed for a mode of action of co-expression of oil synthesising enzyme and oil encapsulating protein in green tissues resulting in elevated photosynthesis with a phenotype of increased seed oil and protein content.

Nutrition trials to assess the nutritional value of PhotoSeed soybean expeller meal compared to conventional soybean expeller meal in chickens were conducted at the University of Illinois (Cristobal et al. 2023) in three experiments to assess nitrogen-corrected true metabolizable energy (TMEn), standardised amino acid digestibility and ileal phosphorus digestibility. PhotoSeed soybean meal contained greater TMEn than conventional soybean meal (3261 vs. 3162 kcal/g DM), there was no difference in amino acid digestibility although there were greater concentrations of some essential amino acids in PhotoSeed soybean meal. Finally, there was no difference in ileal phosphorus digestibility between the two treatments. The study concluded that PhotoSeed soybean meal has greater nutritional value than conventional soybean meal in chickens.

Nutrition trials to assess the nutritional value of PhotoSeed soybean expeller meal compared to conventional soybean expeller meal in pigs were also conducted at the University of Illinois (Cristobal and Stein 2023a, 2023b, 2023c). The studies determined that PhotoSeed soybean expeller meal had greater concentrations of digestible energy than conventional soybean expeller meal. Secondly, PhotoSeed soybean expeller meal had lower digestibility of some amino acids compared to conventional soybean expeller meal, but because there was a greater concentration of amino acids in PhotoSeed soybean expeller meal, the concentration of digestible amino acids was greater. Thirdly, the digestibility of phosphorus was the same for both treatments. This study therefore demonstrated that pig nutritionists could have a lower inclusion rate of PhotoSeed soybean meal in the animal diets, adding value because the animal diet composition will be less expensive.

ZeaKal and Perdue Agribusiness, a company utilising soybean meal in its chicken business formed a partnership in 2022. The results of the nutrition trials support the value of PhotoSeed soybean and as regulatory approval has been obtained it is likely this product will be grown by farmers on the East Coast of the USA in the future.

## Conclusions

The commercialisation of genetically modified nutritionally enhanced food crops have followed crops such as soybean, maize, canola and cotton containing input traits for improved farming efficiency. Part of this reason is the complexity of the traits that lead to enhanced nutritional composition. Many are part of linked metabolic pathways and it has taken significant research to identify approaches to create benefit without detrimental perturbations in plant functions and performance. Another reason is the smaller size or more fragmented seed markets which means fewer investment opportunities for private industry and a requirement for significant government funding. The development of nutritionally enhanced feed and forage crops are even further behind food crops for many of the same reasons and in addition there are challenges with the complex breeding of many obligate outcrossing forages and in forage grasses there have been concerns over pollen from genetically modified species crossing with conventional crops. Often the forage seed companies lack the resources to invest significantly in the research and development needed to use this technology to create value. This has required significant co-investment from governments over long periods.

The examples of HiCT white clover and HME perennial ryegrass illustrate how government and private investment in research can benefit. The perennial ryegrass programme over the last 20 years has had approximately $40 m of investment. From this research, new opportunities have been created including the spin out agricultural biotechnology company ZeaKal that is close to commercial release of PhotoSeed soybean. Clearly further investment is required to progress both these technologies towards commercialisation and into the hands of farmers. At present the focus is on progression in Australia as it has a robust but functional regulatory system in the OGTR and a history of GM crop adoption. It remains to be seen how the current New Zealand government will update the regulations for genetic modification. An outline of policy of the National Party is provided in (Harnessing Biotechnology [date unknown]). An obvious strategy is to progress in Australia and demonstrate the benefits so that New Zealand can be a market follower.
